# ESRRB Facilitates the Conversion of Trophoblast-Like Stem Cells From Induced Pluripotent Stem Cells by Directly Regulating CDX2

**DOI:** 10.3389/fcell.2021.712224

**Published:** 2021-09-20

**Authors:** Shuai Yu, Rui Zhang, Qiaoyan Shen, Zhenshuo Zhu, Juqing Zhang, Xiaolong Wu, Wenxu Zhao, Na Li, Fan Yang, Hongjiang Wei, Jinlian Hua

**Affiliations:** ^1^College of Veterinary Medicine, Shaanxi Centre of Stem Cells Engineering and Technology, Northwest A&F University, Shaanxi, China; ^2^Key Laboratory of Animal Gene Editing and Animal Cloning in Yunnan Province, Yunnan Agricultural University, Kunming, China

**Keywords:** ESRRB, KRT8, Cdx2, induced pluripotent stem cells (iPSCs), trophoblast stem cells (TSCs)

## Abstract

Porcine-induced pluripotent stem cells (piPSCs) could serve as a great model system for human stem cell preclinical research. However, the pluripotency gene network of piPSCs, especially the function for the core transcription factor estrogen-related receptor beta (ESRRB), was poorly understood. Here, we constructed ESRRB-overexpressing piPSCs (ESRRB-piPSCs). Compared with the control piPSCs (CON-piPSCs), the ESRRB-piPSCs showed flat, monolayered colony morphology. Moreover, the ESRRB-piPSCs showed greater chimeric capacity into trophectoderm than CON-piPSCs. We found that ESRRB could directly regulate the expressions of trophoblast stem cell (TSC)-specific markers, including KRT8, KRT18 and CDX2, through binding to their promoter regions. Mutational analysis proved that the N-terminus zinc finger domain is indispensable for ESRRB to regulate the TSC markers. Furthermore, this regulation needs the participation of OCT4. Accordingly, the cooperation between ESRRB and OCT4 facilitates the conversion from pluripotent state to the trophoblast-like state. Our results demonstrated a unique and crucial role of ESRRB in determining piPSCs fate, and shed new light on the molecular mechanism underlying the segregation of embryonic and extra-embryonic lineages.

## Introduction

Pluripotent stem cells (PSCs) are capable of infinite self-renewal and retaining the developmental potential to differentiate into all cell types of an organism (Haider et al., 2018). The maintenance of naïve pluripotent state depends on gene regulatory networks surrounding core transcription factors (Blakeley et al., 2015). Due to the similarity in organ size, physiology, anatomy, and heredity, pigs play a crucial role in human regenerative medicine as an attractive mammalian model (Tamma et al., 2012; Ou et al., 2020). Moreover, human organs generated in transgenic pigs might be able to solve the worldwide organ shortage problem for transplantation (Wu et al., 2017). Hence, it was critical to understand the molecular mechanisms of gene network that regulates the pluripotency and differentiation of porcine embryonic stem cells (pESCs) or porcine-induced PSCs (piPSCs). However, authentic naïve pESCs and piPSCs have not been established. Thus, investigating the pluripotent gene regulatory network of piPSCs was of major importance for future pig PSC-based pre-clinical studies. Certain genes were found co-expressed in embryonic and extra-embryonic cell lineages in porcine embryos based on single-cell RNA-seq (scRNA-seq) studies (Boroviak et al., 2015; Petropoulos et al., 2016; Mohammed et al., 2017; Ramos-Ibeas et al., 2019; Kong et al., 2020). Some of these genes, including estrogen-related receptor beta (ESRRB, also known as NR3B2 or ERR2), were considered to play critical roles in embryonic development and pluripotency maintenance.

Estrogen-related receptor beta is an orphan nuclear receptor with extensive expression profile that regulates energy metabolism in mammals (Festuccia et al., 2018b; Thouennon et al., 2019). ESRRB was also considered as the key transcription factor (TF) that performs specific functions in pluripotent and multipotent cell populations, primordial germ cells (PGCs), as well as in early embryonic development (Benchetrit et al., 2019; Gao H.B. et al., 2019; Okamura et al., 2019; Fan et al., 2020). In mice, ESRRB-knockout (KO) led to the absence of chorion in E7.5 embryos, and abnormalities in early placenta of E8.5 embryos, demonstrating that ESRRB is required for normal placenta development (Luo et al., 1997). In PSCs, ESRRB could reprogram pluripotent cells from primed state to naïve state (Festuccia et al., 2012, 2018a; Atlasi et al., 2019). ESRRB is sufficient to sustain mouse ESC self-renewal in the absence of LIF, and could activate OCT4 transcription and sustain pluripotency in ESCs (Zhang et al., 2008; Iseki et al., 2016). Moreover, ESRRB was a direct target of NANOG in ESCs, and could replace NANOG to maintain the morphology and pluripotency of mouse ESCs (Festuccia et al., 2012; Adachi et al., 2018; Zhang et al., 2018; Sevilla et al., 2021). The evidence demonstrated that ESRRB is an essential member of the network of TFs involved in mammalian embryonic development and pluripotency maintenance.

However, as the key regulator of pluripotency, the role of ESRRB in piPSCs was unclear. Herein, in order to gain insight into the epigenetic regulation of porcine pluripotent gene network, we investigated the expression and regulatory function of ESRRB for the maintenance of pluripotency. We found that ESRRB directly binds and activates a core set of TSC-specific genes, including KRT8, KRT18, CDX2, and BMP4 in the presence of OCT4. Taken together, our results indicate that ESRRB and KRT8 cooperate with OCT4 to facilitate the conversion from pluripotent state to the trophoblast-like state.

## Materials and Methods

### Cell Culture

The piPSCs constructed by Ma et al. (2018) were used in this study. The piPSCs were grown on feeder cells [mouse embryonic fibroblasts (MEFs) treated by mitomycin-C] and maintained in the standard medium as follows (termed LBCS medium): DMEM (Hyclone, United States) supplemented with 15% FBS (VISTECH, VIS59216269, New Zealand), 0.1 mM NEAA (Gibco, United States), 1 mM L-glutaMAX (Gibco), 0.1 mM β-mercaptoethanol (Sigma-Aldrich, United States), 100 U/ml of penicillin, 100 μg/ml of streptomycin, 10 ng/ml of LIF (Sino Biological, China), 10 ng/ml of bFGF (Sino Biological), 3 μM CHIR99021 (MCE, United States), 2 μM SB431542 (Selleck, China), and 4 μg/ml of doxycycline (DOX, Sigma-Aldrich). The piPSCs were subcultured by using TrypLE^TM^ Select (Invitrogen, United States) at a 1:50 ratio every 5–6 days. HEK293T cells used in this study were cultured in DMEM supplemented with 10% FBS, 0.1 mM NEAA (Gibco, United States), 1 mM L-glutaMAX (Gibco), 0.1 mM β-mercaptoethanol (Sigma-Aldrich, United States), 100 U/ml of penicillin, and 100 μg/ml of streptomycin.

### Vector Construction

The plasmid backbones used in this study were derived from pCDH-CMV-MCS-EF1-puromycin and pCDH-U6-MCS-EF1-puromycin. The following expressing constructs (Table 1) were generated using NovoRec plus One-step PCR Cloning Kit (Novoprotein, China).

**TABLE 1:** The DNA constructs used in this experiment.

	Construct name	Vector source
Lentivirus vectors	pCDH-CMV-MCS-EF1-GreenPuro	SBI, Mountain View, United States
	EF1-ESRRB-P2A- puromycin	This work
	EF1-3 × FLAG-ESRRB-P2A-puromycin	This work
	EF1-3 × FLAG-ESRRB-N Terminus-P2A-puromycin	This work
	EF1-3 × FLAG-ESRRB-C Terminus-P2A-puromycin	This work
	Rosa26-EF1-OCT4-SOX2-P2A-puromycin (OS)	This work
	Rosa26-EF1-OCT4-ESRRB-P2A-puromycin (OE)	This work
	Rosa26-EF1-ESRRB-SOX2-P2A-puromycin (ES)	This work
	Rosa26-EF1-GFP-P2A-puromycin (GFP)	This work
	shRNA-NC	This work
	shRNA-ESRRB	This work
	PGL3-basic vector	Promega, E1751
	PGL3-basic CDX2-promoter	This work
	PGL3-basic KRT8-promoter	This work
	173-ESRRB	This work
	173-ESRRB N-terminus	This work
	173-ESRRB C-terminus	This work
	155-SOX2	This work
	155-KRT8	This work
	173-negative control	Addgene 22010
	155-negative control	Addgene 22011

### Overexpression Vector Construction

To investigate the function of ESRRB, the genes, such as ESRRB and its domain, were PCR amplified from porcine blastocysts and then subcloned into EF1-MCS-T2A-puromycin lentiviral vector and EF1-3FLAG-MCS-T2A-puromycin lentiviral vector.

In order to explore the combinatorial effect of pluripotent genes with ESRRB, the coexpression constructs of OCT4–SOX2 (OS), OCT4–ESRRB (OE), ESRRB–SOX2 (ES), and GFP were designed to insert into porcine Rosa26 locus using the CRISPR/Cas9-mediated knock-in system. The donor plasmids were constructed as previously described (Zhang et al., 2019).

### shRNA Vector Construction

In this study, we used knockdown technology to detect the function of ESRRB. shRNA against ESRRB was designed using the online shRNA design tool from ThermoFisher^1^ and cloned into PCDH-U6-MCS-EF1-GFP-T2A-puromycin vector. The potential off-target effect was filtered out using BLAST against porcine genome. The shRNA virus was then infected to piPSCs, and that with knockdown efficiencies above 60% were used in this study (Table 2).

**TABLE 2:** shRNA primers used in this experiment.

Primer name	Forward sequence	Reverse sequence
shESRRB	gatccGTGCGAGTACATGCTCAACGTCAAGA GCGTTGAGCATGTACTCGCACTTTTTTg	aattcAAAAAAGTGCGAGTACATGCTCAAC GCTCTTGACGTTGAGCATGTACTCGCACg

### Luciferase Vector Construction

To verify the binding sequences of ESRRB and its domain, the porcine gene promoter of CDX2 was PCR amplified from total genomic DNA and subcloned into the PGL3-basic vector (Promega).

### Lentivirus Packaging and Transduction

Lentiviral vectors were transfected into HEK 293T cells using PEI (PolyScience, United States) according to the instructions of the manufacturer (Zhu et al., 2021a). The medium containing viral particles was collected and filtered through 0.45-μm filters after 24 h (Millipore, United States). An equal ratio of viral particles was mixed and used to infect piPSCs with polybrene for 12 h. The infected piPSCs were then plated on feeder-coated six-well plate and cultured by the piPSC induction medium for 5–7 days. The piPSCs were then screened with 10 μg/ml of puromycin for 12 h, and the puromycin-resistant cell lines were established.

### RNA Extraction, Reverse Transcription and qRT-PCR Detection

Total RNA was extracted by using the RNAiso Plus reagent (TaKaRa, China). Then the cDNA was synthesized by reverse transcription (RT) using the PrimeSript^TM^ RT reagent kit (Tiangen, China) according to its protocol. The quantitative RT-PCR (qRT-PCR) reaction system was 20 μl in volume: 10 μl of SYBR^®^ Premix Ex Taq II (Tiangen), 1 μl of cDNA, 0.5 μl of PCR Forward Primer (10 μmol/L), 0.5 μl of PCR Reverse Primer (10 μmol/L), with RNase-free water added to a total volume of 20 μl. The specific primers of PCR for genes used in this study are shown in Table 3.

**TABLE 3:** Quantitative RT (qRT)-PCR primers used in this experiment.

Primer name	Forward sequence	Reverse sequence
ESRRB	AAATACAAGCGACGGCTGGA	GGAGGCATGGCGTAGAGTTT
endo-OCT4	CTTCACCACCCTGTACTCCTCG	CAGGCTTCTCTCCCTAGCTCAC
endo-SOX2	ATGTCCCAGCACTACCAGAGCG	CTTACTCTCCTCCCATTTCCCTCT
exo-pOCT4	TCACTTCACCACCCTGTACT	CACCTGCTTGCTTTAGCAGA
exo-pSOX2	CAGACTTCACATGTCCCAGCACTA	CGGGATTCTCCTCCACGTCA
endo-KLF4	GAGGGAAGACCAGAATCCCTTGTA	TAGAACCAAGACTCACCAAGCACC
endo-ESRRB	GACGGGCAAGTTGCTGCTGACG	CGGTCCATCCATTTGTCTGTCC
KLF2	CGAGGCTTGATGCCTTGTGA	GCCCACCTGCCTTCCTATTT
ZIC3	TGTGCACACCTCGGACAAG	TATAGCGGGTGGAGTGGAAGA
LIN28A	GAAGTCTGCTAAGGGCTTGGAATC	TGTCTCCCTTGGATCTGCGTTT
CDX2	TGTGCGAGTGGATGCGGAAG	CTCCGAATGGTGATGTAGCGACTG
KRT8	TCAGATTTCCGACACCTCCG	AATCTCCGTCTTCGTGCGAC
KRT18	TGCTGATGACTTCAGAGTCAAG	TTACTTCCTCCTCGTGGTTC
GATA2	AGTATCTCCTGACCCCAGCA	ACTGCCGCTTTCCATCTTCA
GATA3	ACGTCCTGTGCAAACTGTCA	TCGGTTTCTGGTCTGGATGC
TEAD4	CGCCTCAGCCTTCCACAATA	CGGCTGGACAGTGTAGGTTT
NES	TTCCAAGGCTTCTCTCAGCATCT	GCTCTTCAGAAAGGCTGGCATA
PAX6	CAGAGAAGACAGGCCAGCAA	GGCAGAGCACTGTAGGTGTT
ZIC1	CCAACGTGGTTAATGGGCAG	TAGTGCTCTGAACGGGGA
GFAP	CTCAACGTTAAGCTGGCCCT	CAGGCTGGTTTCTCGGATCT
GBX2	GCCAAATGGAAACGGGTGAA	TCTAATGGCGAACCTGCTGA
DES	GGCTCAGTACGAGACCATCG	GCATCGATCTCGCAGGTGTA
FGF8	AACTCTACAGTCGCACCAGC	ATGTAGAGGCCCGTTTCAGC
BMP4	TTGTTCAGGATTGGCTGTCAAGA	CTAGCAGGACTTGGCATAAT AAAACG
PECAM	GACGTGGAGTACACGGAAGTG	ATCTGTTTTCCACTAAATCAGGGT
ACTIN	GCAAGGACCTCTACGCCAACA	TGGAGGCGCGATGATCTTG

### Alkaline Phosphatase Staining

The pluripotency of piPSCs were detected by alkaline phosphatase (AP) staining reagent according to the instructions of the manufacturer (Wu et al., 2021; Zhu et al., 2021a). In brief, the cells were fixed with 4% paraformaldehyde (Sangon Biotech) in PBS for 15 min at room temperature and washed twice with PBS. Then the cells were incubated with AP staining reagent [consisting of 1.0 mg/ml of Fast Red TR and 0.4 mg/ml of Naphthol AS-MX (Sigma-Aldrich)] in 0.1 M Tris Buffer at room temperature. The AP-positive colonies were shown in red color after 20 min of incubation. Finally, the images were captured by a fluorescent phase-contrast microscope (Nikon, Japan).

### Immunofluorescence

The cells were fixed with 4% paraformaldehyde for 15 min and then washed twice in PBS, permeabilized with 0.1% Triton X-100 for 10 min and blocked by 10% FBS for 1 h at room temperature. Afterward, the cells were incubated with primary antibodies at 4°C for 12–16 h. Then the cells were incubated for 1 h with their appropriate secondary antibodies at 37°C. The cell nucleus was counterstained with Hoechst33342 (1:1,000; Beyotime, China), and the images were captured with a phase-contrast microscope (Nikon). The specific primary antibodies used in this study are shown in Table 4.

**TABLE 4:** Antibodies used in this experiment.

Antibody	Source	Catalog number
OCT4	Santa Cruz	sc-5279
SOX2	Proteintech	66411-1-Ig
ESRRB	Proteintech	22644-1-Ig
AFP	Proteintech	14550-1-AP
DESMIN	Proteintech	16520-1-AP
TUBB3	Proteintech	66375-1-Ig
CDX2	Bioss	bs-6694R
KRT8	Proteintech	14550-1-AP
ACTIN	Proteintech	66411-1-Ig

### Western Blot Analysis

The cells were lysed by cold RIPA buffer (Beyotime) for 30 min on ice, mixed with 5× SDS-PAGE loading buffer (Beyotime), and heated at 100°C for 10 min. The protein lysates were separated by 8–12% SDS-PAGE and transferred to polyvinylidene difluoride (PVDF) membranes by Trans-Blot SD Cell and Systems (Bio-Rad) for 50 min at 15 V. After blocking with 5% non-fat milk in TBST buffer solution for 2 h, the PVDF membrane was incubated with the special primary antibody (Table 4). After incubation with the HRP-conjugated secondary antibody (Life Technology), signals were measured using ECL reagents (Tanon, China) and the Chemiluminescent Imaging System (Tanon).

### EdU Staining

The proliferation ability of the cells was detected by Cell-Light EdU Apollo643 *in vitro* kit (RIB BIO, China) according to the instructions of the manufacturer. In brief, the cells were incubated with solution A for 20–30 min at 37°C, then fixed with 4% paraformaldehyde in PBS for 15 min at room temperature, and sequentially incubated with solution Apollo643 for 20 min and Hoechst33342 for 5 min. The images were captured with the fluorescent phase-contrast microscope (Nikon).

### Apoptosis Analysis

The apoptosis analysis was performed by Annexin V-FITC/PI Apoptosis Detection Kit (Vazyme, China) according to the instructions of the manufacturer. In brief, the cell suspension was washed twice with precooled PBS and centrifuged at 1,000 × *g* for 5 min, followed by adding 100 μl of 1× binding buffer and blowing gently to make a single-cell suspension. Then 5 μl of Annexin V-FITC and 5 μl of PI staining solution were added and incubated for 10 min. Finally, 400 μl of 1× binding buffer was added and mixed gently. The images were captured with a fluorescent phase-contrast microscope (Nikon).

### Embryoid Body Formation and Spontaneous Differentiation

The piPSCs were digested by TryPLE select^TM^, cultured in low-cell-binding dishes with LBCS medium for 1 day, and then transferred to the DMEM medium including 15% FBS (Gibco), 0.1 mM NEAA, 1 mM L-glutaMAX, 100 U/ml of penicillin, 100 μg/ml of streptomycin, and 0.1 mM β-mercaptoethanol for 6 days. Embryoid bodies (EBs) were transferred to 0.1% (w/v) gelatin-coated dishes and cultured in DMEM medium for 7 days. The medium was changed every day. Morphology observation and immunofluorescence were used to evaluate the differentiation of the three germ layers as reported previously (Shen et al., 2019).

### Pig Oocytes and Parthenogenetic Embryo Activation

Porcine ovaries were obtained from Hongteng slaughterhouse (Yunnan, China) and were maintained at 25–30°C during transportation to the laboratory. The selection standard for ovarian follicles includes a diameter between 3 and 6 mm and cumulus–oocyte complexes comprising more than three layers of cumulus cells based on published protocols (Wei et al., 2013; Niu et al., 2017). Oocytes were cultured *in vitro* in a maturation medium at 38.5°C under 5% CO_2_.

The matured oocytes were used to develop into parthenogenetic embryos by electrical activation (Wei et al., 2013). In brief, oocytes overlaid with activating treatment were immediately activated with 150 V DC for 100 μs using an electro cell fusion generator (LF201; Nepa Gene) and then incubated in PZM3 medium for 6 h at 38.5°C in 5% CO_2_. After 6 h, the oocytes were washed with IVC medium and transferred to fresh PZM3 medium at 38.5°C in 5% CO_2_. Cleavage and blastocyst formation were evaluated on days 2 and 6. The development rate of each stage (two-, four-cell, and morula) of embryos was evaluated at 30, 48, and 96 h of IVC medium after activation, respectively.

### Microinjection of piPSCs Into Eight-Cell Embryos

piPSCs were cultured in LBCS medium for 2 days, dissociated by Accutase for 3–5 min. Then the cells were collected into 1.5-ml tubes, washed with PBS, and resuspended in M2 medium (Wei et al., 2013; Niu et al., 2017). Five to 10 piPSCs were injected into the eight-cell embryos, and the experiment was repeated three times. After the injected eight-cell embryos developed into blastocysts, some of the late blastocysts were used for immunohistochemistry to identify the chimerism by the donor cells. CDX2 was used as a TE marker.

### Luciferase Assay

First, the HEK293T cells were seeded into 48-well plates. At 70% confluence, the cells were transfected by 0.5 μg of ESRRB or its domain-expressing plasmids, 0.5 μg of promoter reporter plasmids, and 0.01 μg of pRL-TK controls with PEI for 12 h. Then the cells were lysed by cell lysis solution for 15 min at RT after post-transfection at 48 h. Finally, luciferase activity was measured using a dual-luciferase detection kit (Beyotime, RG027), and the data were collected by a Hamamatsu BHP9504 Luminometer (Vigorous, China) according to the recommendations of the manufacturer.

### RNA-seq Analysis

Total RNA of RNA samples was isolated from cell pellets by TRIZOL reagent (TaKaRa), and RNA-seq library was generated according to the recommendations of the manufacturer. Paired-end 150-bp sequencing was performed on DNBSEQ (Illumina) at BGI Technology Corporation (China). Clean reads were aligned to pig genome Ssc11.1, and the expression level was normalized as RPKM with gene annotation file. Differential expression genes and functional enrichment for Gene Ontology (GO) and KEGG were plotted by http://www.bioinformatics.com.cn, an online platform for data analysis and visualization. The raw data were uploaded in GEO datasets (GSE180057).

### Chromatin Immunoprecipitation-seq Analysis

ESRRB-overexpressing piPSCs were crosslinked with formaldehyde and sonicated to 200–1,000 bp. Then the sonicated chromatin was immunoprecipitated with the antibody FLAG. The DNA fragments were quantified by agarose electrophoresis using Qubit 2.0 and sequenced on Illumina HiSeq2500 PE150 by SEQHEALTH Technology Corporation (China). The clean reads were mapped to pig genome Ssc11.1 (NCBI) using STRA software (version 2.5.3a) with default parameters. The biological replicates were then pooled together for each group, and downstream analyses were performed. The signal intensity for each sample was calculated, which was defined as ±2 kb around the transcription start site (TSS). The OCT4 chromatin immunoprecipitation (ChIP)-seq data were based on the study of Zhu et al. (2021b). Annotation and visualization of ChIP peak coverage over the chromosomes were conducted using the ChIPseeker package in R (version 1.18.0) (Yu et al., 2015). The raw data were uploaded in GEO datasets (GSE180056).

### Immunoprecipitation-Mass Spectrometry Analysis

The cells were dissociated, pelleted, and cytoplasm were removed by incubation in hypotonic buffer. The nuclei were pelleted, washed, and permeabilized with 0.1% *n*-octyl-β-D-glucopyranoside. Nuclei were digested with MNase at a concentration of 2 U/μl at 37°C for 5 min. Nucleosomes with associated proteins were extracted in IP buffer, incubated with anti-FLAG magnetic beads overnight at 4°C, washed five times with IP buffer, and eluted with FLAG peptide at 150 ng/ml. Immunoprecipitants were subjected to SDS-PAGE and probed with indicated antibodies or detected by silver staining according to the protocol of the manufacturer. Then the gel pieces were sent to PTM Biolab Technology Corporation (China). The resulting IP-mass spectrometry data were processed using Proteome Discoverer 1.3. Tandem mass spectra were searched against the UniPort database. Trypsin/P (or other enzymes if any) was specified as cleavage enzymes allowing up to two missing cleavages. The mass error was set to 10 ppm for precursor ions and 0.02 Da for fragment ions. Carbamidomethyl on Cys was specified as fixed modification, and oxidation on Met was specified as variable modification. Peptide confidence was set at high, and peptide ion score was set > 20. The OCT4 IP-mass spectrometry data were based on the study of Zhu et al. (2021b), and the ESRRB IP-mass spectrometry data were uploaded in PRIDE datasets (PXD027539).

### Statistical Analyses

All experiments were repeated at least three times. All data were analyzed using SPSS 18.0 (SPSS Inc., Chicago, IL, United States) and were expressed as the means ± standard errors (SE). ANOVA with Tukey’s HSD *post hoc test* was applied to multigroup comparisons, whereas Student’s *t*-test was used for the two-group comparisons. When the *p*-value was less than 0.05, the data were considered statistically significant.

## Results

### Estrogen-Related Receptor Beta Affected the Self-Renew and Differentiation Potential of Porcine-Induced Pluripotent Stem Cells

Recent scRNA-seq of porcine early embryos showed that ESRRB was highly expressed in morula, ICM, and trophectoderm (TE) cells, and OCT4 was highly expressed in morula, ICM, TE, and late blastocysts (Supplementary Figure 1A). We used the porcine parthenogenetic embryos to detect the expression level of ESRRB. As shown in Supplementary Figure 1B, the expression of ESRRB started at the eight-cell stage and could be seen in the morula, ICM, and TE cells, consistent with the scRNA-seq results. This indicated that ESRRB may play a role both in PSCs and TE cells.

**FIGURE 1:**
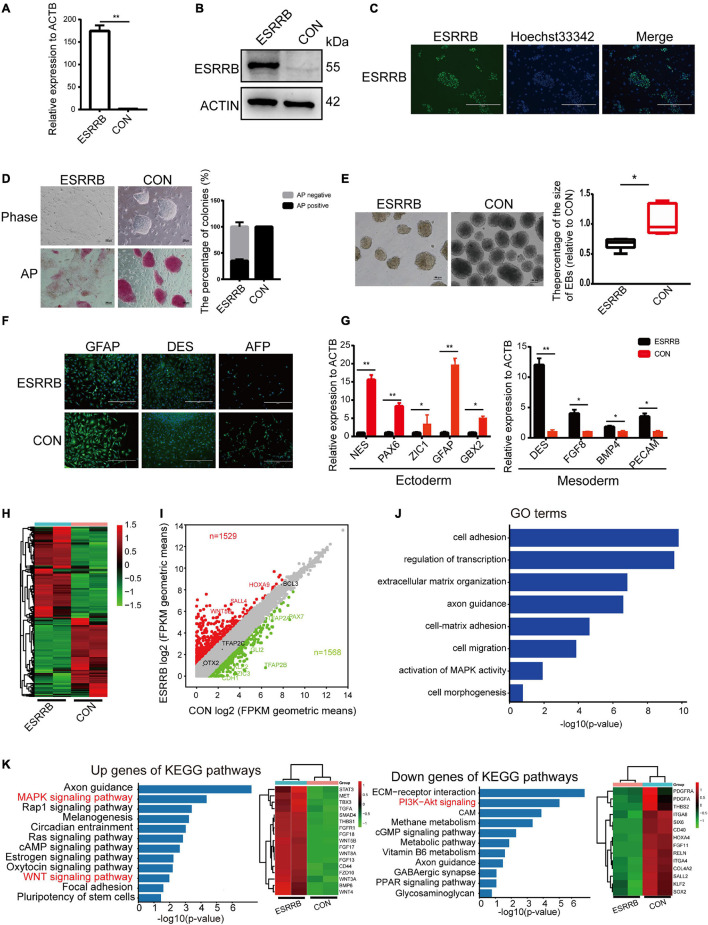
Estrogen-related receptor beta (ESRRB) affected the self-renew and differentiation potential of porcine-induced pluripotent stem cells (piPSCs). **(A)** Quantitative RT (qRT)-PCR analysis of ESRRB in the control (CON) and ESRRB-piPSCs; **p < 0.001. **(B)** Western blot analysis of the ESRRB level in the CON- and ESRRB-piPSCs. **(C)** Immunofluorescence analysis of ESRRB in the ESRRB piPSCs. The scale bar represents 400 μm. **(D)** Representative image of bright field and alkaline phosphatase (AP) stained colonies in the control (CON) and ESRRB piPSCs. The scale bar represents 100 μm. The quantitative analysis of AP-positive colonies is shown by histogram (the CON group was the control). **(E)** The embryoid body (EB) formation in the CON- and ESRRB-piPSCs. The scale bar represents 100 μm. The quantitative analysis is shown by histogram. **(F)** The immunofluorescence staining of three germ layers in the CON- and ESRRB-piPSCs. The scale bar represents 400 μm; **(G)** qRT-PCR analysis of three germ layers markers in the EBs derived from CON- and ESRRB-piPSCs; *p < 0.05 and **p < 0.001. **(H)** The heatmap of CON- and ESRRB-piPSCs. **(I)** The volcano plot showing the number of up- or downregulated differentially expressed (DE) genes in the CON- and ESRRB-piPSCs detected by RNA-seq analysis. *n* = 2 independent experiments. **(J)** The Gene Ontology (GO) enrichment terms of biological process in the CON- and ESRRB-piPSCs. **(K)** the KEGG enrichment for up- and downregulated genes in the CON- and ESRRB-piPSCs; the right heatmap was the genes enriched into the signaling pathways.

To investigate this potential role of ESRRB in piPSCs, we generated ESRRB-overexpressing piPSCs using lentivirus vector (marked as ESRRB-piPSCs) in which ESRRB expression was enhanced. qRT-PCR and Western blot analyses revealed that the expression of ESRRB was significantly greater in ESRRB-piPSCs than that of CON-piPSCs (Figures 1A,B). Immunofluorescence showed that the ESRRB was observed in the nucleus of ESRRB-piPSCs, in accordance with its role as transcription factor (Figure 1C). Notably, ESRRB-piPSCs formed flat colonies and showed attenuated or even negative AP staining (Figure 1D). Furthermore, we examined the differentiation capacity of ESRRB-piPSCs. Both CON- and ESRRB-piPSCs could form EBs and differentiate into three germ layer cells [positive for the ectoderm (GFAP), mesoderm (DES), and endoderm (AFP) markers] (Figures 1E,F and Supplementary Figure 1C). However, the EBs derived from ESRRB-piPSCs were significantly smaller than those of the CON-piPSCs (Figure 1E). The difference in EB size should be driven by the expression of differentiation markers. qRT-PCR analysis showed that the expressions of ectoderm markers, such as *NES*, *PAX6*, *ZIC1*, *GBX2*, and *GFAP*, were significantly decreased in ESRRB-EBs when compared with the CON-EBs. However, the expression levels of mesoderm markers, such as *DES*, *FGF8*, *BMP4*, and *PECAM*, were significantly increased in ESRRB-EBs (Figure 1G). The endoderm markers were not significantly decreased in ESRRB-EBs when compared with the CON-EBs (Supplementary Figure 1D). These results predicted that ESRRB could affect pluripotency and differentiation of piPSCs.

We further analyzed the apoptosis and proliferation potential of ESRRB-piPSCs. Annexin V/PI staining showed that there was no significant difference in apoptosis (Supplementary Figure 2A). However, the percentage of EdU-positive cells in the CON-piPSCs was greater than that of ESRRB-piPSCs, indicating that ESRRB overexpression could decrease the piPSC proliferation (Supplementary Figure 2B). The expression levels of proliferation-related genes in CON- and ESRRB-piPSCs was then evaluated. We found that CDK1 and PCNA were highly expressed in ESRRB-piPSCs than in CON-piPSCs (Supplementary Figure 2C). Besides, the ChIP-seq analysis revealed a significant rise in ESRRB in adjacent genomic regions of CCND1 and CCND2 in ESRRB-piPSCs. These data suggested a direct regulatory function by ESRRB for the proliferation-related genes (Supplementary Figure 2D).

RNA-seq was performed to provide a snapshot of the transcriptional dynamics in order to investigate the effect of ESRRB overexpression in piPSCs. The heatmap analysis (Figure 1H) revealed that the global transcriptional profile of the ESRRB-piPSC group was distant from that of CON-piPSCs. Differentially expressed (DE) genes between ESRRB- and CON-piPSCs (1,529 up versus 1,568 down) were analyzed by volcano plots (Figure 1I). In addition, GO biological process terms such as cell adhesion, regulation of transcription, extracellular matrix organization, axon guidance, cell migration, regulation of MAP kinase activity, and cell morphogenesis were enriched from the DE genes (Figure 1J). The KEGG pathway enrichment analysis using the list of upregulated DE genes in ESRRB-piPSCs showed enriched signaling pathways, including MAPK, Rap1, WNT, and pluripotency of stem cell signaling pathways (Figure 1K). Also, the list of DE genes downregulated in ESRRB-piPSCs had enriched KEGG pathways, including PI3K-Akt, cGMP, and ECM receptor signaling pathways (Figure 1K). These results indicated that ESRRB overexpression substantially changed the cellular network status of the piPSCs.

### Overexpression of Estrogen-Related Receptor Beta Resulted in Greater Chimeric Ability Into Trophectoderm

**FIGURE 2:**
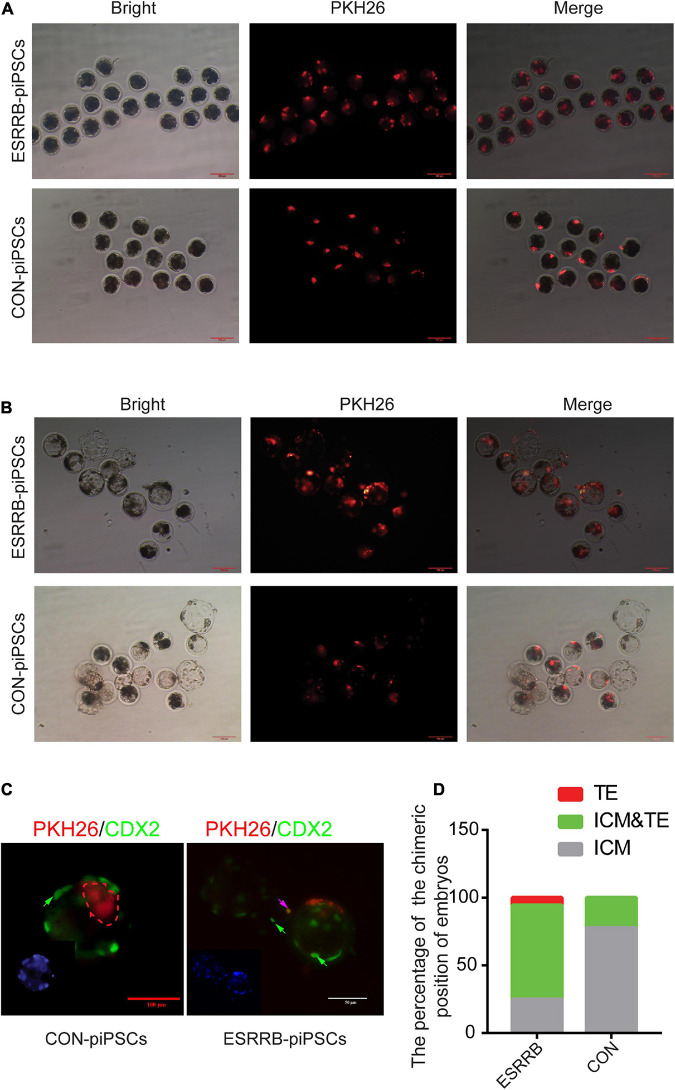
Overexpression of ESRRB in greater chimeric ability into trophoblast. **(A)** The localization of injected CON- and ESRRB-piPSCs in early eight-cell pig embryo; the scale bar represents 100 μm. **(B)** The localization of injected CON- and ESRRB-piPSCs chimeric embryos after 72 h culture *in vitro*; the scale bar represents 100 μm. **(C)** The localization of injected ESRRB-piPSC chimeric blastocysts *in vitro*. The TE was indicated by immunofluorescence of CDX2. The nuclei were Hoechst33342 stained, and the donor cells were indicated by PKH26 staining in red fluorescence. The green arrow represents the CDX2-positive cells, and the pink arrow represents CDX2 and PKH26-double positive cells. The scale bar represents 50 μm. **(D)** The quantitative analysis of contributing cell localization in embryos is shown by histogram.

Since our results indicated that ESRRB may affect the pluripotency and differentiation ability of piPSCs, the chimeric ability of ESRRB-piPSCs was evaluated using pig parthenogenetic embryos as recipients. Five to 10 ESRRB- or CON-piPSCs marked by PKH26 were injected into eight-cell porcine embryos in two groups (Figure 2A). The cells with PKH26 (shown in red fluorescence) was observed in the porcine embryos after injection (Figure 2A). When developed into blastocysts, ESRRB- and CON-piPSCs could be detected with a high survival rate (Figure 2B). The chimeric rates of porcine embryos transplanted with ESRRB- and CON-piPSCs were 54.54 and 56.69%, respectively. Herein, CDX2 was used as the TSC marker to detect the position of ESRRB- and CON-piPSC in chimeric blastocysts (Figure 2C). Notably, ESRRB-piPSCs could be detected in both TE and ICM after 72 h *in vitro* culture post-injection, and the CON-piPSCs could be detected mainly in ICM (Figure 2B and Table 5). Among all ESRRB-piPSC chimeric blastocysts, the rate of contribution to ICM was 25.0%, the rate of contribution to ICM and TE was 69.4%, and the rate of contribution to TE was 5.6%. By contrast, for CON-piPSCs, the rate of contribution to ICM was 77.8%, the rate of contribution to ICM and TE was 22.2%, and the rate of contribution to TE was 0% (*p* < 0.05) (Figure 2D and Table 5). In summary, overexpression of ESRRB endowed piPSCs with the greater ability to develop into trophectoderm parts.

**TABLE 5:** The rate of contributing cell localization in embryos.

Groups	Embryo age	Injecting embryos	Chimeric embryos	Chimeric rate	Chimeric into ICM	The rate of chimeric into ICM	Chimeric into ICM and TE	The rate of chimeric into ICM and TE	Chimeric into TE	The rate of chimeric into TE
ESRRB-piPSCs	8-Cell	66	36	54.54%	9	25.00%	25	69.40%	2	5.60%
CON-piPSCs	8-Cell	79	45	56.96%	35	77.80%	10	22.20%	0	0

### CDX2 Was a Direct Target of Estrogen-Related Receptor Beta

**FIGURE 3:**
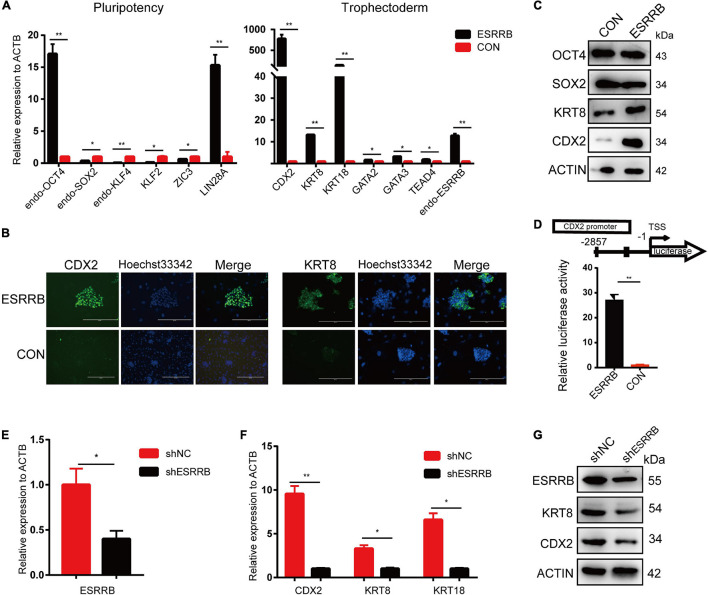
CDX2 was the direct target of ESRRB. **(A)** qRT-PCR analysis of the expressions of the pluripotent stem cell (PSC) and trophoblast (TSC) markers in the CON- and ESRRB-piPSCs. *p < 0.05 and **p < 0.001. **(B)** Immunofluorescence analysis of CDX2 and KRT8 in the ESRRB-piPSCs. The scale bar represents 200 μm. **(C)** Western blot analysis of OCT4, SOX2, KRT8, and CDX2 of the CON- and ESRRB-piPSCs. **(D)** Promoter-luciferase assays for the CDX2-binding region of ESRRB and negative control (NC). NC, empty vector. Data are from three biological replicates and are shown as the mean ± SEM. **p < 0.001. **(E)** qRT-PCR analysis of the expression of ESRRB after ESRRB knockdown in the ESRRB-piPSCs. *p < 0.05. **(F)** qRT-PCR analysis of the expressions of *CDX2*, *KRT8*, and *KRT18* after ESRRB knockdown in the ESRRB-piPSCs. *p < 0.05. **(G)** Western blot analysis of the expression of ESRRB, CDX2, and KRT8 in the ESRRB piPSCs after ESRRB knockdown.

We then investigated the expression levels of key genes related to pluripotency and trophoblast development in ESRRB-piPSCs. As shown in Figure 3A, the expression levels of pluripotency markers, such as *endo-SOX2*, *endo-KLF4*, *KLF2*, and *ZIC3*, were downregulated, and the expression levels of *endo-OCT4* and *LIN28A* were upregulated in ESRRB-piPSCs compared with those in CON-piPSCs. On the other side, the expressions of TSC markers, such as *CDX2*, *KRT8*, *KRT18*, *GATA2*, *GATA3*, *TEAD4*, and *endo-ESRRB* were upregulated in ESRRB-piPSCs compared with CON-piPSCs, consistent with the RNA-seq results. Immunofluorescence and Western blot analyses revealed that overexpression of ESRRB resulted in upregulation of CDX2 and KRT8 (Figures 3B,C). The expression of CDX2 was found in the nucleus, and the expression of KRT8 was in the cytoplasm and plasma membrane (Figure 3B). Moreover, Western blot revealed that the expression of SOX2 was remarkably downregulated in ESRRB-piPSCs (Figure 3C). We next used several different approaches to investigate the regulation of CDX2 by ESRRB.

First, to investigate the impact of inhibitors in ESRRB-piPSC culture medium, we changed the culture condition seriatim (Supplementary Figure 3A). The results showed that the colony morphology of ESRRB-piPSCs is dependent on CHIR99021 and SB431542, while LIF and bFGF slightly affected cell proliferation (Supplementary Figure 3B). The qRT-PCR analysis of the ESRRB and CDX2 under different treatments revealed that CHIR99021 and SB431542 had an only minor effect on the expression of CDX2 (Supplementary Figure 3C) compared with the highly elevated CDX2 by ESRRB overexpression (Figure 3A). This result indicated that the phenotype of ESRRB-piPSCs was not related to the culture system.

Second, to further determine whether ESRRB is a functional activator of CDX2, ESRRB-expression plasmid was co-transfected with reporter plasmid with the expression of luciferase under the control of CDX2 promoter region (pGL3-CDX2). A 27-fold increase in the CDX2 promoter reporter activity was found relative to the control group (Figure 3D). To investigate whether ESRRB downregulation attenuates CDX2 expression, we constructed lentiviral constructs expressing pig ESRRB-specific shRNA sequences (shESRRB). Stable knockdown (50–60%) piPSC colonies by shRNA of ESRRB transcript was observed following drug selection (Figure 3E and Supplementary Figure 3D). As expected, the *CDX2*, *KRT18*, and *KRT8* transcripts were decreased in shESRRB cells when compared with scramble control (Figure 3F). The Western blot showed that the expression of CDX2 and KRT8 was remarkably downregulated in shESRRB piPSCs compared with the control group, consistent with the qRT-PCR results (Figure 3G). Collectively, these results demonstrated that CDX2 is positively regulated and activated by ESRRB in piPSCs.

### N-Terminus of Estrogen-Related Receptor Beta Was the Main Domain Regulating CDX2

**FIGURE 4:**
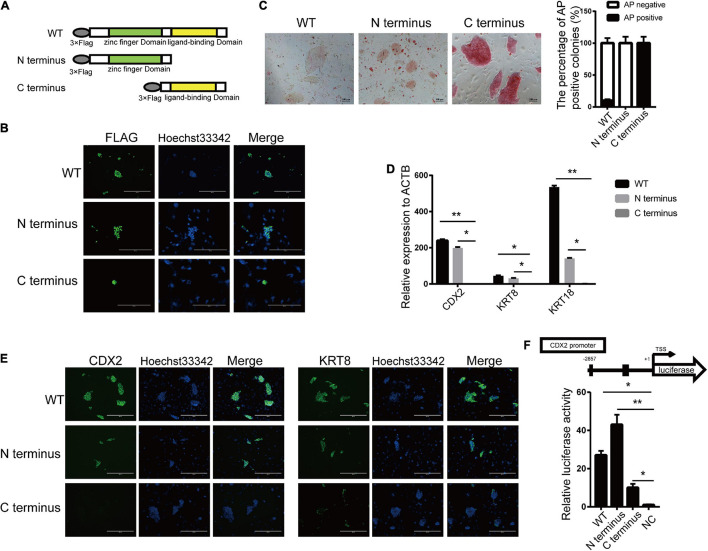
N-terminal of ESRRB was the main domain in regulating CDX2. **(A)** A schematic illustration of the different domains of ESRRB with a FLAG epitope. **(B)** Immunofluorescence analysis of FLAG in the three constructed cell lines. **(C)** Representative image of AP-stained colonies after 5 days of clonal growth of the three constructed cell lines. The scale bar represents 100 μm. The quantitative analysis of AP-positive rate is shown by histogram. **(D)** qRT-PCR analysis of the CDX2, KRT8, and KRT18 expressions in the three constructed piPSC lines. *p < 0.05 and **p < 0.001. **(E)** Immunofluorescence analysis of CDX2 and KRT8 in the three constructed cell lines. The scale bar represents 200 μm. **(F)** Promoter-luciferase assays for the CDX2-binding region of ESRRB-WT, N-terminus, and C-terminus. NC, empty vector. Data are from three biological replicates and are shown as mean ± SEM.

Estrogen-related receptor beta contains two distinct domains, the zinc finger domain at the N-terminus and the ligand-binding domain at the C-terminus. ESRRB was formed as a hexametric in solution via the ligand-binding domain while bound directly to DNA via zinc finger domain (Festuccia et al., 2018b). To define the domain that acts on pluripotency and differentiation regulation for ESRRB, we generated expression constructs encoding full-length (WT) and two truncated ESRRB proteins lacking the zinc finger domain (C-terminus) or the ligand-binding domain (N-terminus) (Figure 4A). These FLAG-tagged recombinant ESRRB constructs were overexpressed in piPSCs. The subcellular localization of FLAG-tagged full-length and truncated proteins of ESRRB was found in the nucleus of piPSCs (Figure 4B), and Western blot showed that FLAG-tagged proteins could be detected in these three piPSC groups (Figure 4B and Supplementary Figure 4A). Notably, overexpression of the N-terminus ESRRB in piPSCs promoted formation of flat colonies with weakened AP staining comparable with the WT group (Figure 4C). On the contrary, overexpression of the C-terminal ESRRB in piPSCs promoted formation of tight colonies with stronger AP staining when compared with the WT group (Figure 4C). For the trophoblast markers, qRT-PCR showed that the expressions of KRT8 and CDX2 were high in WT and the N-terminus ESRRB groups, and low in the C-terminus group (Figure 4D). Immunofluorescence results showed that the KRT8 and CDX2 were particularly expressed in the WT and N-terminus ESRRB groups but not in the C-terminus group, consistent with the qRT-PCR results (Figure 4E). To further determine which ESRRB domain is the functional activator of CDX2 promoter, these three vectors were cotransfected together with the reporter vector with the expression of luciferase controlled by the CDX2 promoter region. Under these conditions, we observed a 27-fold increase reporter activity in the ESRRB WT group, a 45-fold increase in the N-terminus group, and a 12-fold increase in the C-terminus group relative to the negative control (Figure 4F). Collectively, these results signified that CDX2 was positively regulated and directly activated by the N-terminus of *ESRRB* in piPSCs.

### CDX2 Expression Was Modulated by Both OCT4 and Estrogen-Related Receptor Beta

**FIGURE 5:**
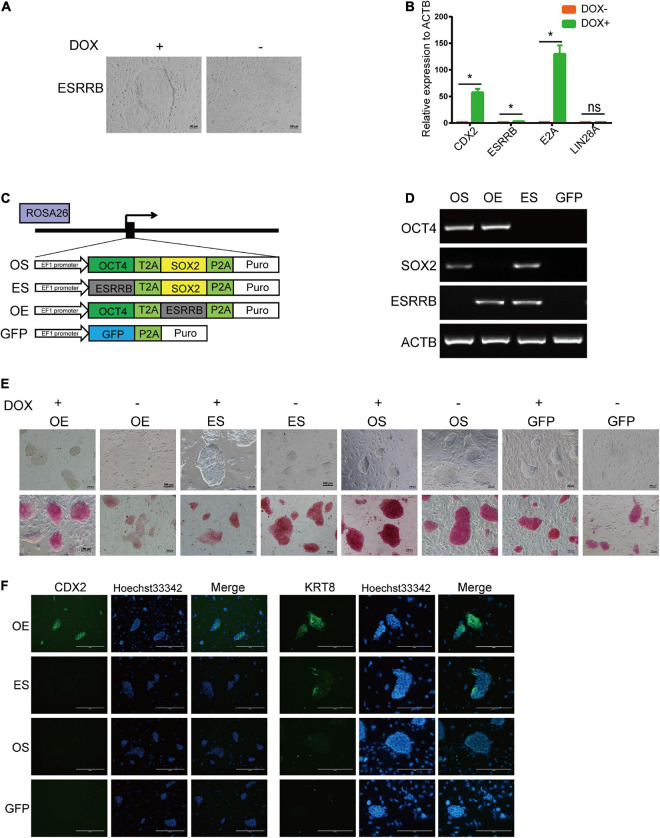
CDX2 expression was modulated by both OCT4 and ESRRB. **(A)** Representative image of colonies after doxycycline (DOX) withdrawal. *p < 0.05; **(B)** qRT-PCR analysis of *CDX2*, *ESRRB*, *E2A*, and *LIN28A* in the DOX and DOX-withdrawal groups. **(C)** A schematic illustration of different combination of ESRRB and OCT4 (OE), ESRRB and SOX2 (ES), OCT4 and SOX2 (OS), and GFP constructs to be inserted into the ROSA26 site. **(D)** RT-PCR analysis of the expressions of *ESRRB* and porcine-specific exogenous *OCT4*, *SOX2*, and *ACTB* in the OS, OE, ES, and GFP cells. **(E)** Representative image of AP-stained colonies of the four constructed cell lines with DOX or after DOX withdrawal after 5 days. The scale bar represents 100 μm. **(F)** Immunofluorescence analysis of CDX2 and KRT8 in the four constructed cell lines without DOX. The scale bar represents 200 μm.

The self-renewal of piPSCs was dependent on the sustained expression of exogenous genes, which were induced by DOX. The withdrawal of DOX in culture medium resulted in the downregulation of exogenous as well as endogenous pluripotent genes. We asked whether the other pluripotent genes also modulate the expression of TSC markers. As shown in Figure 5A and Supplementary Figure 4B, the morphology of ESRRB-piPSCs became deteriorated, and colonies could not be formed without DOX. qRT-PCR results showed that the expression of *CDX2* and pluripotent genes, such as *OCT4* and *SOX2*, significantly declined with DOX withdrawal, which confirmed that the CDX2 was not only regulated by ESRRB, but also by other pluripotent genes (Figure 5B).

To validate the potential relationship of ESRRB and other pluripotent genes, we constructed OCT4-ESRRB (OE), OCT4-SOX2 (OS), ESRRB-SOX2 (ES), and GFP overexpression vectors and inserted them into the ROSA26 location of piPSCs (Figure 5C). RT-PCR was used to identify the cell lines that were successfully constructed by using ESRRB prime and the porcine-specific exogenous OCT4 and SOX2 primers (Figure 5D). In these groups, the OS and GFP groups were AP positive, while the OE and ES groups had attenuated AP staining in the presence of DOX (Figure 5E). Upon withdrawal of DOX, the OS group could be maintained in AP-positive colonies and could not detect the expression of CDX2, while the ES and GFP groups lost expression of CDX2, and the colonies could not be maintained (Figures 5E,F). However, the OE group had weakened AP-positive colonies, with the expression of CDX2 unaffected by DOX withdrawal (Figures 5E,F). These results indicated that the expression of CDX2 also depends on OCT4 expression. Taken together, these data demonstrated that CDX2 is modulated by both OCT4 and ESRRB.

**FIGURE 6:**
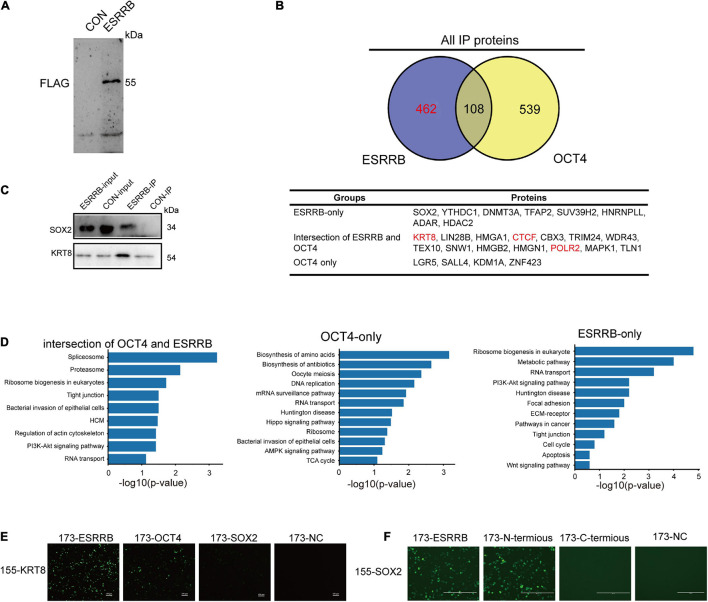
Cooperative binding of core OCT4 and ESRRB to regulate CDX2. **(A)** Immunoprecipitation of ESRRB was validated using Western blot analysis. The piPSCs overexpressing 3FLAG-ESRRB were used to purify ESRRB-associated proteins. **(B)** Venn diagrams showing the overlap of OCT4- and ESRRB-associated proteins determined by mass spectrometry. The table lists some representative proteins of each section. **(C)** The correlation between ESRRB and OCT4 were validated by Western blot analysis. **(D)** The KEGG enrichment of ESRRB or OCT4-associated protein. **(E)** Representative image of bimolecular fluorescence complementation of ESRRB, SOX2, OCT4, and KRT8. The scale bar represents 200 μm. **(F)** Representative image of bimolecular fluorescence complementation of ESRRB, its domain vectors, and SOX2. The scale bar represents 200 μm.

To further understand the molecular mechanism of OCT4, ESRRB, and CDX2, we applied IP-mass spectrometry to directly investigate the proteins related to OCT4 and ESRRB (Figure 6A and Supplementary Figure 4C). The mass spectrometry results showed that there were 570 proteins directly bound by ESRRB and 647 proteins directly bound by OCT4. Among them there were 108 common proteins interacting with both ESRRB and OCT4, including KRT8, CTCF, SNW1, TRIM24, and HMGA2 (Figure 6B). Further analysis of ESRRB revealed a direct relationship between ESRRB with SOX2 and KRT8 (Figure 6C). The OCT4-interacting proteins were enriched with establishment of protein localization to telomere and positive regulation of telomerase activity. The ESRRB-interacting proteins were enriched with positive regulation of the PI3K-Akt signaling pathway and metabolic pathway process (Figure 6D). In addition, we found that the proteins interacting with both the ESRRB and OCT4 were enriched with PI3K-Akt signaling pathway, tight junction, and RNA transport. We also used the bimolecular fluorescence complementary (BiFC) technique to verify the direct binding between KRT8 with OCT4 and ESRRB. As shown in Figure 6E, recombinant KRT8 could tightly associate with ESRRB to produce GFP fluorescence, consistent with the co-IP result, while KRT8 could also associate with OCT4, although to a lesser extent than ESRRB. Moreover, we found that recombinant SOX2 is tightly associated with the full length and N-terminus, but not C-terminus, of ESRRB (Figure 6F). Taken together, our data indicate that ESRRB protein works with OCT4 to directly activate downstream targets including CDX2 and KRT8.

### Estrogen-Related Receptor Beta Bound CDX2 and KRT8 in Porcine-Induced Pluripotent Stem Cells

**FIGURE 7:**
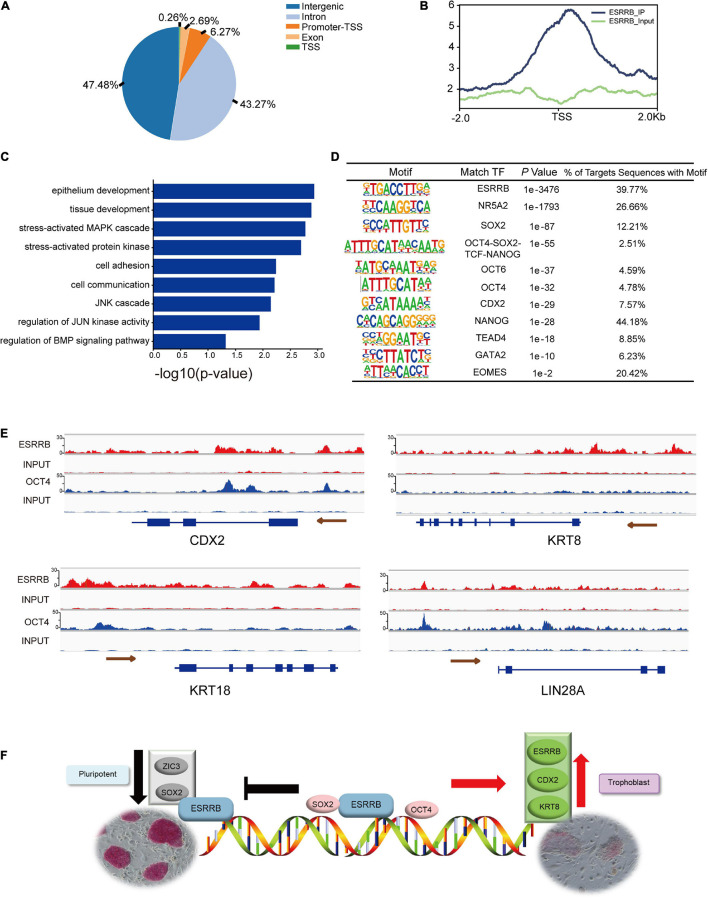
Chromatin immunoprecipitation (ChIP)-seq analysis for genes targeted by ESRRB. **(A)** Fraction of ESRRB-FLAG binding sites within various regions of the genome. **(B)** The profiles of peak signals of ESRRB-FLAG. Signals are shown for 2.0 kb up- and downstream of the transcription start site (TSS). **(C)** The GO enrichment of ESRRB peak correlative genes. **(D)** The motifs of ESRRB were identified. **(E)** ChIP-seq analysis of ESRRB marks at pluripotent and TSC gene loci in ESRRB-piPSCs using IGV. The size of the peak represents the degree of enrichment. **(F)** The model for action for OCT4, KRT8, CDX2, and ESRRB during pluripotency and trophoblast conversion.

The protein–protein interaction proved that both OCT4 and ESRRB could be interacted by KRT8, then we further used ChIP-seq analysis to detect direct downstream target genes of ESRRB. The FLAG-ESRRB-piPSCs (F-piPSCs) lines were successfully constructed so that we could use an anti-FLAG antibody to conduct ChIP-seq. The phenotype of the F-piPSCs was the same as ESRRB-piPSCs. As shown in Figure 7A, the peak FLAG-antibody captured was mainly associated with intragenic intron and intergenic regions. This correlated with the role of ESRRB as a transcription regulator. A total of 19,694 peak calling and their proximity to NCBI-designated genes (5,533 genes) were detected (Figure 7B and Supplementary Figure 5A). GO biological process terms such as epithelium development, cell adhesion, regulation of transcription, extracellular matrix organization, axon guidance, cell migration, regulation of JUN signaling pathway, and regulation of BMP signaling pathway were enriched from the peak-associated genes enriched by ESRRB (Figure 7C). Then we combined the results of the RNA-seq and ChIP-seq and found that 884 ESRRB directly targeted genes and showed differential expression. The KEGG pathway enrichment analysis using the upregulated genes directly targeted by ESRRB showed enriched KEGG pathways including Ras, MAPK, and focal adhesion (Supplementary Figure 5B), while the downregulated genes targeted by ESRRB showed enriched KEGG pathways including PI3K-AKT, Camp, and ECM receptor signaling pathways (Supplementary Figure 5C).

Further motif analysis revealed that many DNA motifs identified from ESRRB ChIP-seq data were actual targets of pluripotency genes (SOX2, OCT4, and NANOG) and TE markers (CDX2, TEAD4, and GATA2) (Figure 7D). Moreover, the enhancer and promoter regions upstream of CDX2 and LIN28A were directly bound by OCT4 and ESRRB, the upstream regions of KRT8 and KRT18 were directly bound by ESRRB, while the gene body region of CDX2 was directly bound by OCT4 (Figure 7E). These results demonstrated that ESRRB and OCT4 directly regulate the expression of CDX2, and ESRRB directly regulates KRT8 in piPSCs. In summary, ESRRB and OCT4 could facilitate the conversion from the pluripotent state to the trophoblast-like stem cell state through regulating key TSC gene expression (Figure 7F).

## Discussion

The molecular mechanisms underlying porcine embryo development had yet to be fully understood. This work provided evidence for a novel role of ESRRB as gatekeeper in the development of porcine embryos. Herein, we demonstrated that the ectopic expression of ESRRB was sufficient to overcome the lineage barrier between embryonic and extraembryonic cells inducing stable TSC-like cells, which could maintain TSC morphology during different passages. In addition, the regulation of TSC markers (CDX2 and KRT8) was assumed to occur by the ESRRB and OCT4 protein because deletion of either one stopped TSC marker expression. Our study therefore established ESRRB and OCT4 as the critical mediators in the trophectoderm differentiation of piPSCs.

Estrogen-related receptor beta could be coexpressed in the late morula and early blastocyst of pig preimplantation embryos according to scRNA-seq studies, which suggested that ESRRB plays a critical role in the functional maintenance of porcine ESCs and TSCs (Ramos-Ibeas et al., 2019; Kong et al., 2020). We demonstrated that overexpression of ESRRB could trigger piPSCs differentiation into TSC-like cells. For the first time, we established a chromatin accessibility landscape in iPSCs, and identified transcription factor-binding motifs at ESRRB-binding regions that become accessible to both pluripotency and TSC regulators during embryo development. We envision that more pluripotent states, including naïve, primed, and formative states, could be investigated in the future to understand comprehensively the different regulatory roles of porcine ESRRB.

It was well established that ESRRB drove the transition between primed- and naïve-state pluripotency (Festuccia et al., 2012, 2016; Adachi et al., 2018; Hao et al., 2019). In line with this evidence, ESRRB could achieve highly efficient conversion of EpiSC to naïve pluripotency (Festuccia et al., 2016, 2018a). ESRRB was not unique in this regard, and reexpression of other preimplantation pluripotency factors downregulated in EpiSCs, including KLF4, PRDM14 in conjunction with KLF2, NR5A1, NR5A2, GBX2, and TFCP2L1 (Iseki et al., 2016; Collier et al., 2017; Saunders et al., 2017), can also drive this conversion with variable efficiencies. However, direct ESRRB overexpression induced EpiSCs reprogramming with an efficiency that greatly surpassed that of NANOG. In this study, we found that ESRRB was also a marker of porcine expanded PSCs, and direct ESRRB overexpression promoted the transformation of TSC-like cells from primed piPSCs. This is different from the results from mice (Castel et al., 2020).

We explored why direct ESRRB overexpression leads to different results in primed-state mouse and porcine PSCs. Changing the culture condition seriatim showed that the culture conditions played an insignificant role for the function of ESRRB. Then we speculated that it might be due to the difference of gene network between mouse and porcine TSCs. SOX2 and ESRRB are expressed in mouse TSCs, in which the expression of OCT4 is absent (Benchetrit et al., 2019; Gao H.B. et al., 2019). However, OCT4 and ESRRB are both expressed in porcine TSCs, while the expression of SOX2 is absent. Here, we found that ESRRB could decrease the expression of SOX2. This could be one mechanism for ESRRB to promote the transformation of TSCs from piPSCs. Otherwise, in mouse TSCs, ESRRB was a pivotal regulator in the transcriptional network of TSCs, and forced expression of ESRRB partially blocked the rapid differentiation of TSCs in the absence of Fgf4, and ESRRB-deficient TSCs lost the ability of hemorrhagic lesion formation *in vivo* (Palamadai Krishnan, 2015; Zhang et al., 2018; Gao H.B. et al., 2019; Okamura et al., 2019). These results predicted that ESRRB could regulate the TSC markers, consistent with the results in our study.

ESRRB has the unique ability to shape the regulatory architecture of iPSCs, acting at key gene enhancers and serving as a direct connection between pluripotency TFs and the basal transcriptional machinery. ESRRB further safeguards the stability of the pluripotency network, facilitating its transmission through mitosis via its bookmarking activity, and directly favors a metabolic regime associated with naive pluripotency (Sone et al., 2017). Hence, ESRRB may occupy a central role in the gene regulatory network associated with pluripotency by coordinating and stabilizing the expression of cell identity and metabolic genes through its mitotic bookmarking activity (Adachi et al., 2018). Proteomic analyses have underscored that ESRRB represents a key node of the pluripotency interactome. ESRRB interacts with a number of other pluripotency regulators, such as OCT4, SOX2, and TFCP2L1 (Acampora et al., 2017; Wang et al., 2019). In a previous study, the protein–protein interactions identified an ESRRB-SOX2 composite motif, which was considered as the second most prevalent compound motif after the canonical OCT4-SOX2 consensus (Engelen et al., 2015). In our study, we identified that there is an interaction between N-terminus zinc finger domain of ESRRB and SOX2 in piPSCs. We further discovered that ESRRB-regulated CDX2 expression is mediated by its zinc finger domain, which was never verified before.

In summary, ESRRB combined with OCT4 and KRT8 regulates TSC-specific markers to facilitate the conversion from the pluripotent state to trophoblastic-like state. The zinc finger domain of ESRRB plays a critical role in this process. Our study illustrated a role of ESRRB serving as the key intermediary connecting the network transition process between the piPSCs and TSCs.

## Data Availability Statement

The datasets presented in this study can be found in online repositories. The names of the repository/repositories and accession number(s) can be found below: NCBI (accession: GSE180058).

## Ethics Statement

The animal study was reviewed and approved by the Animal Care and Use Committee of Northwest A&F University.

## Author Contributions

SY and JH designed the research. SY performed the main experiments. RZ, QS, ZZ, JZ, XW, WZ, NL, FY, and HW helped with the experiments. SY wrote the manuscript. All the authors have read and agreed to the published version of the manuscript.

## Conflict of Interest

The authors declare that the research was conducted in the absence of any commercial or financial relationships that could be construed as a potential conflict of interest.

## Publisher’s Note

All claims expressed in this article are solely those of the authors and do not necessarily represent those of their affiliated organizations, or those of the publisher, the editors and the reviewers. Any product that may be evaluated in this article, or claim that may be made by its manufacturer, is not guaranteed or endorsed by the publisher.

## Footnotes

1
http://rnaidesigner.thermofisher.com/rnaiexpress/

## Supplementary Material

Supplementary Figure 1 The expression pattern of ESRRB in pig embryos. **(A)** Box Plot of the expression of selected pluripotency and lineage specified genes based on the data from Ramos-Ibeas et al. (2019). EB, early blastocyst; LB, late blastocyst; Sph, spherical embryo; EPI, epiblast; HYPO, hypoblast; ICM, inner cell mass; TE, trophectoderm; **(B)** the expression of ESRRB in early porcine embryos. **(B)** The expression level of ESRRB in porcine pre-implantation embryos; **(C)** Immunofluorescence analysis of GFAP and DES. Intensity values along the indicated path were obtained using ImageJ and shown by line chart. The red line represents CON-piPSCs and the blue line represents ESRRB-piPSCs; **(D)** qRT-PCR analysis of endoderm markers in the CON- and ESRRB-piPSCs.

Supplementary Figure 2 ESRRB affected cell proliferation via CCND1 and CCND2. **(A)** Representative images of cells stained with PI and Annexin V after 5 days of culture of the CON and ESRRB-piPSCs; **(B)** representative images of cells in the CON- and ESRRB-piPSCs and exposed to EdU in order to evaluate proliferation. *n* = 3 independent experiments. The quantitative analysis of EdU positive colonies was shown by histogram (the CON group was the control); **(C)** the expression of proliferation-associated genes in CON- and ESRRB-piPSCs; **(D)** ChIP-seq analysis of ESRRB marked at CCND1 and at CCND2 loci using IGV.

Supplementary Figure 3 The expression of CDX2 was inconspicuously affected by CHIR99021 and SB431542. **(A)** The model for the function of CHIR and SB; **(B)** ESRRB piPSCs were cultured in media with or without LIF, bFGF, CHIR99021 and SB431542; **(C)** qRT-PCR analysis of ESRRB and CDX2 in the indicated conditions. The 15% FBS and LB groups could not survive thus there were no samples could be collected; **(D)** the phenotype of ESRRB-piPSCs after ESRRB knockdown.

Supplementary Figure 4 The effect of DOX on ESRRB piPSCs. **(A)** Western blot analysis of FLAG in the ESRRB-WT, N-terminus and C-terminus domain cell lines; **(B)** the phenotype of ESRRB-piPSCs after using different doses of DOX; **(C)** ESRRB-interacting proteins were visualized by Coomassie brilliant blue staining.

Supplementary Figure 5 The combined analysis of the RNA-seq and ChIP-seq of ESRRB. **(A)** Distribution of ESRRB-FLAG ChIP-seq signal on both sides of transcription start sites; **(B)** results from upregulated genes directly targeted by ESRRB; **(C)** results from downregulated genes directly targeted by ESRRB.
