# Supplementary figures and images for: ESRRB Facilitates the Conversion of Trophoblast-Like Stem Cells From Induced Pluripotent Stem Cells by Directly Regulating CDX2
Source: Front Cell Dev Biol. 2021 Sep 20;9:712224. doi: 10.3389/fcell.2021.712224 (PMC8488167; doi:10.3389/fcell.2021.712224)

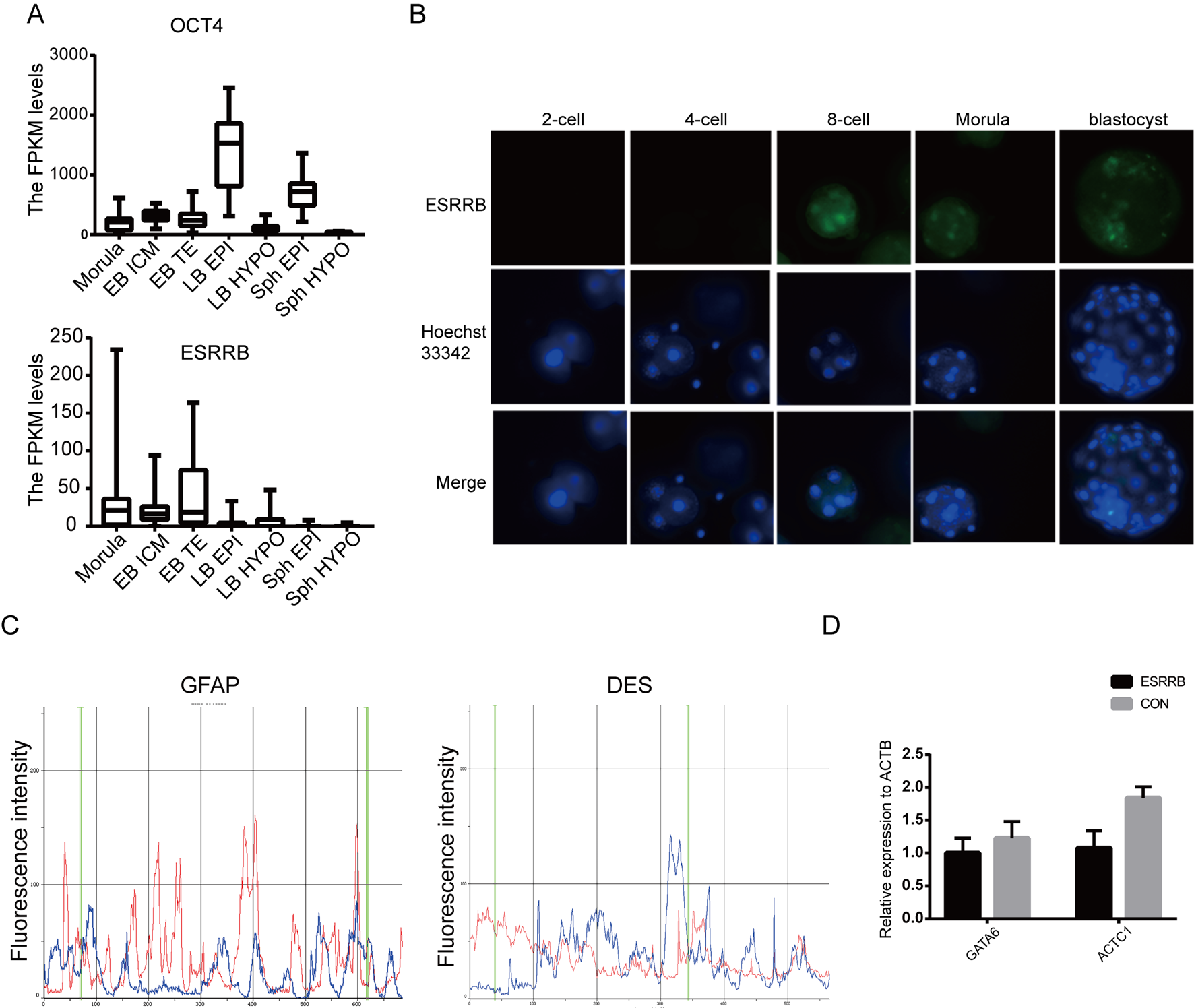

Supplement: Supplementary Figure 1 — The expression pattern of ESRRB in pig embryos. (A) Box Plot of the expression of selected pluripotency and lineage specified genes based on the data from Ramos-Ibeas et al. (2019). EB, early blastocyst; LB, late blastocyst; Sph, spherical embryo; EPI, epiblast; HYPO, hypoblast; ICM, inner cell mass; TE, trophectoderm; (B) the expression of ESRRB in early porcine embryos. (B) The expression level of ESRRB in porcine pre-implantation embryos; (C) Immunofluorescence analysis of GFAP and DES. Intensity values along the indicated path were obtained using ImageJ and shown by line chart. The red line represents CON-piPSCs and the blue line represents ESRRB-piPSCs; (D) qRT-PCR analysis of endoderm markers in the CON- and ESRRB-piPSCs. [file Image_1.TIF]

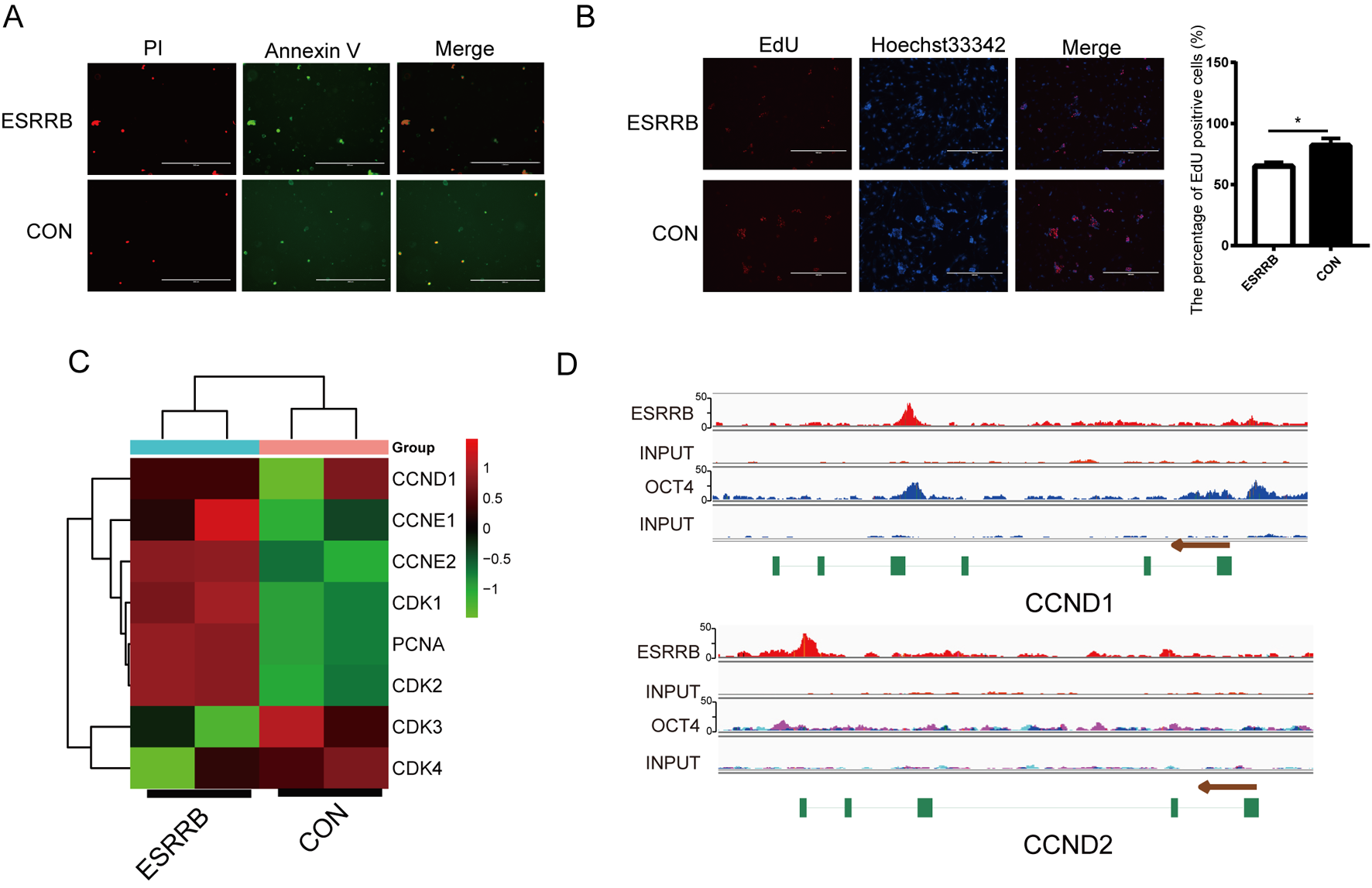

Supplement: Supplementary Figure 2 — ESRRB affected cell proliferation via CCND1 and CCND2. (A) Representative images of cells stained with PI and Annexin V after 5 days of culture of the CON and ESRRB-piPSCs; (B) representative images of cells in the CON- and ESRRB-piPSCs and exposed to EdU in order to evaluate proliferation. n = 3 independent experiments. The quantitative analysis of EdU positive colonies was shown by histogram (the CON group was the control); (C) the expression of proliferation-associated genes in CON- and ESRRB-piPSCs; (D) ChIP-seq analysis of ESRRB marked at CCND1 and at CCND2 loci using IGV. [file Image_2.TIF]

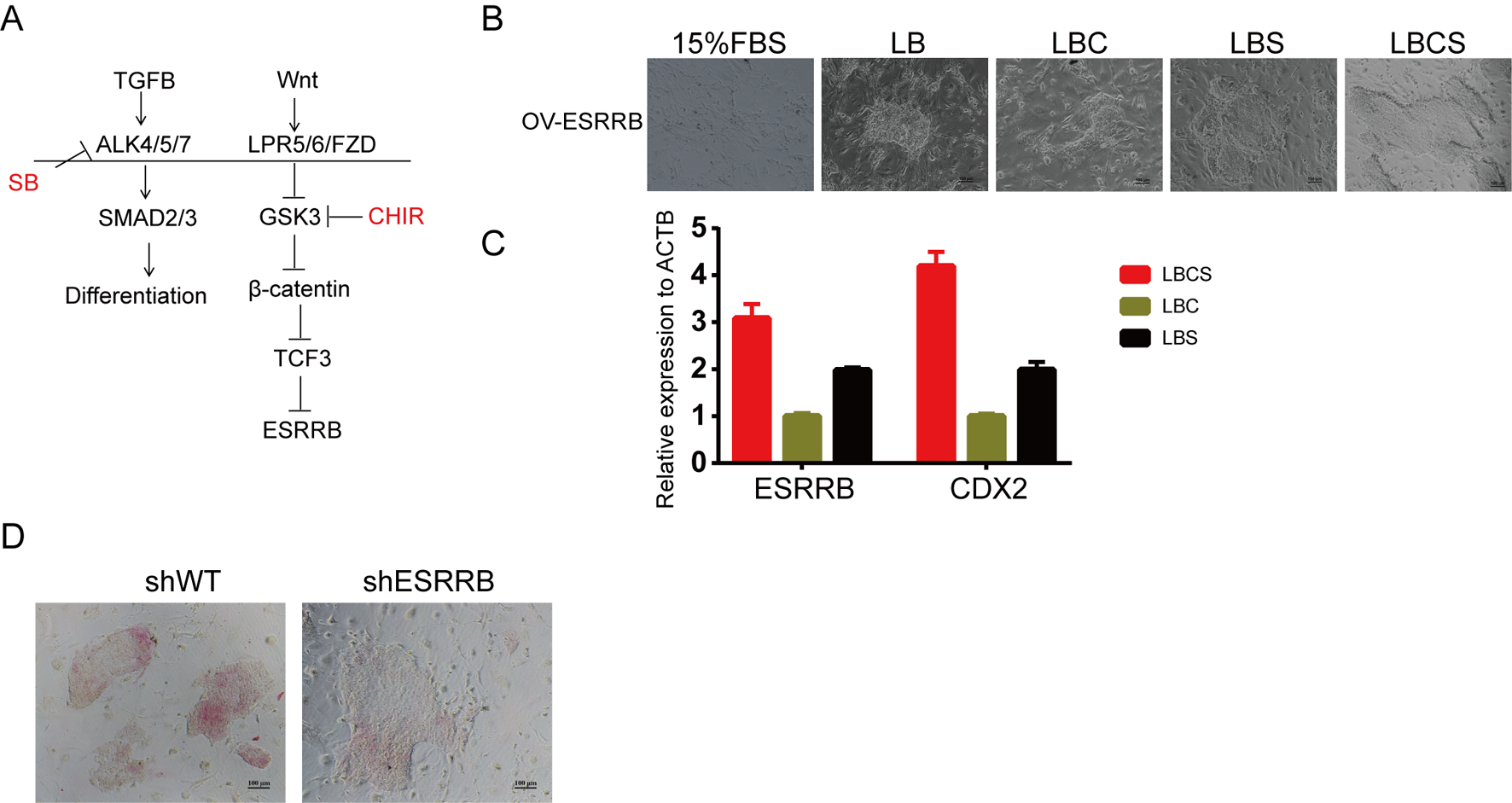

Supplement: Supplementary Figure 3 — The expression of CDX2 was inconspicuously affected by CHIR99021 and SB431542. (A) The model for the function of CHIR and SB; (B) ESRRB piPSCs were cultured in media with or without LIF, bFGF, CHIR99021 and SB431542; (C) qRT-PCR analysis of ESRRB and CDX2 in the indicated conditions. The 15% FBS and LB groups could not survive thus there were no samples could be collected; (D) the phenotype of ESRRB-piPSCs after ESRRB knockdown. [file Image_3.TIF]

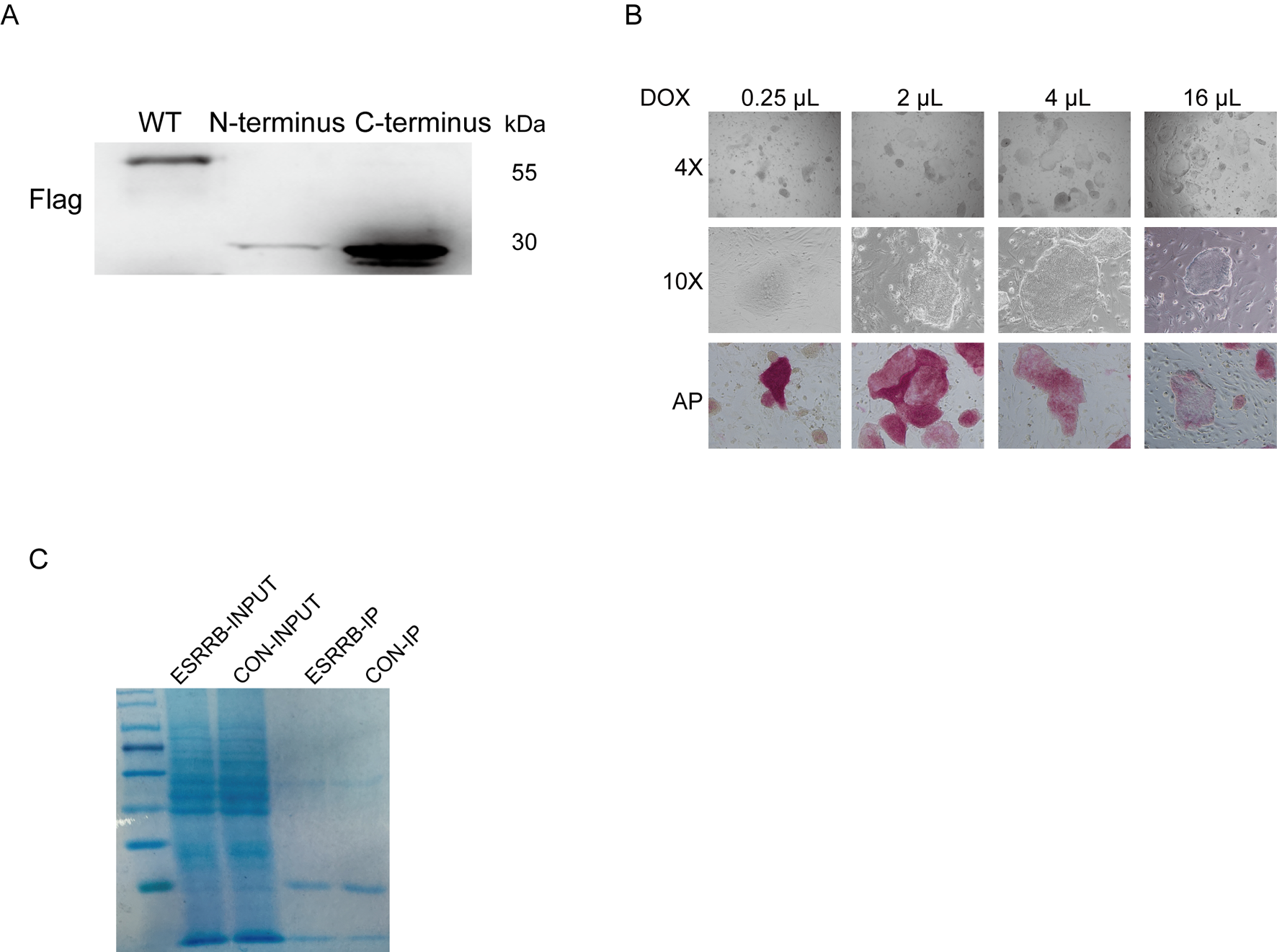

Supplement: Supplementary Figure 4 — The effect of DOX on ESRRB piPSCs. (A) Western blot analysis of FLAG in the ESRRB-WT, N-terminus and C-terminus domain cell lines; (B) the phenotype of ESRRB-piPSCs after using different doses of DOX; (C) ESRRB-interacting proteins were visualized by Coomassie brilliant blue staining. [file Image_4.TIF]

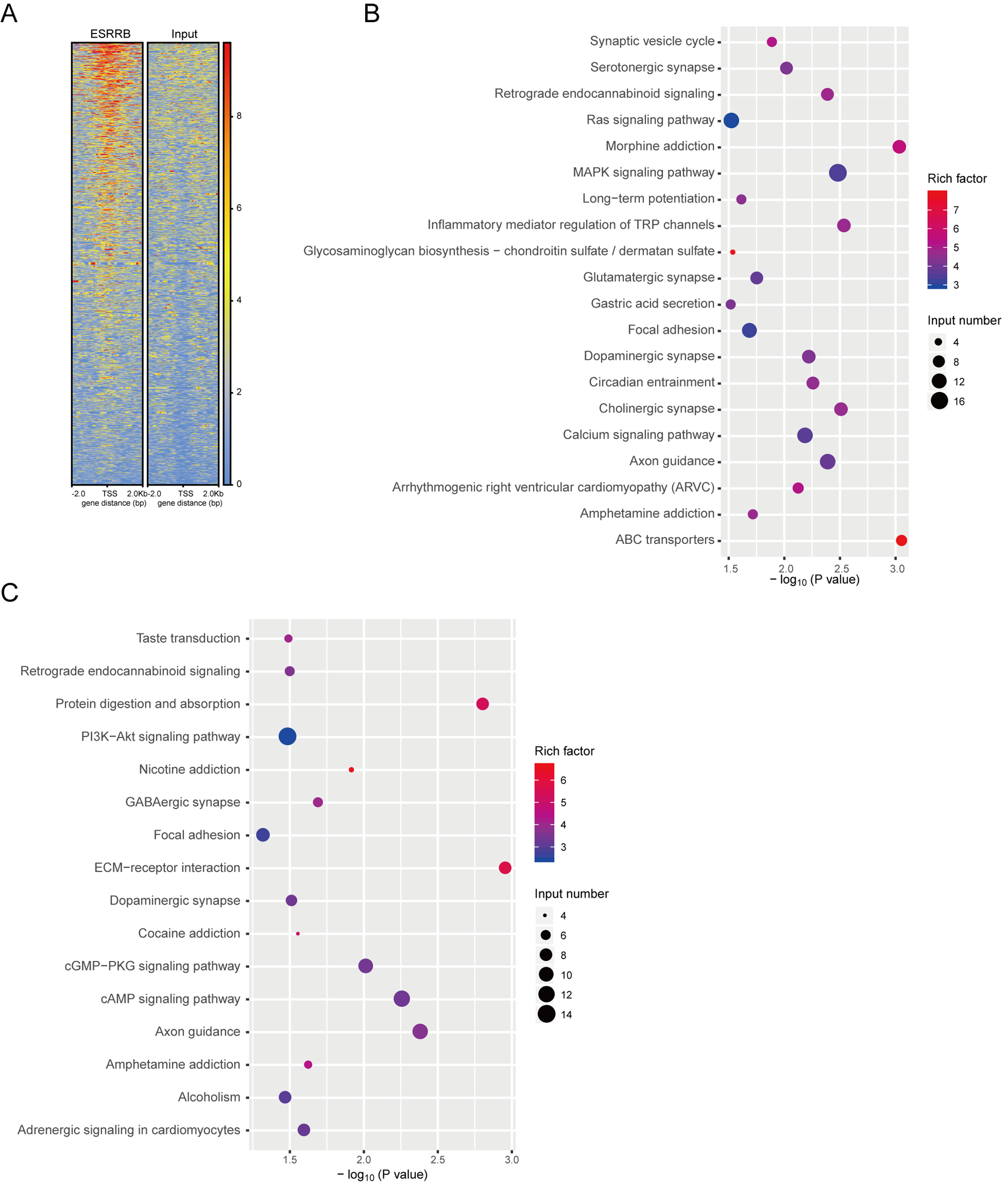

Supplement: Supplementary Figure 5 — The combined analysis of the RNA-seq and ChIP-seq of ESRRB. (A) Distribution of ESRRB-FLAG ChIP-seq signal on both sides of transcription start sites; (B) results from upregulated genes directly targeted by ESRRB; (C) results from downregulated genes directly targeted by ESRRB. [file Image_5.TIF]
